# Severe Post-Viral Polymyositis after COVID-19 in Childhood: A Case Report and Literature Review

**DOI:** 10.3390/children11081011

**Published:** 2024-08-20

**Authors:** Jurgita Marciulynaite, Rima Sileikiene, Ausra Snipaitiene

**Affiliations:** Department of Pediatrics, Medical Academy, Lithuanian University of Health Sciences, Eiveniu Str., 50161 Kaunas, Lithuania; rima.sileikiene@lsmu.lt (R.S.); ausra.snipaitiene@lsmu.lt (A.S.)

**Keywords:** myositis, polymyositis, post-viral, post-COVID, SARS-CoV-2, children, pediatric

## Abstract

Polymyositis is a rarely reported complication of COVID-19 illness, especially in children. Molecular mimicry may be a cause of hyperactivated autoimmunity, leading to various clinical manifestations, including myopathies. Symptoms vary from mild muscle weakness to severe rhabdomyolysis. We review the literature on post-COVID myositis and report a case of severe polymyositis in a 7-year-old boy, following undefined viral infection 3 weeks before the onset of muscle pain. Patient’s condition deteriorated from physical activity-associated pain in the lower limbs to severe muscle weakness leading to dysphagia and mechanical ventilation. As antibodies against SARS-CoV-2 were detected and other possible conditions causing myositis were excluded, the diagnosis of post-COVID polymyositis was considered as the most likely. The patient was treated with high doses of methylprednisolone and cyclophosphamide, resulting in improvement. Although COVID-19 is becoming a seasonal disease, the infection itself and post-viral disorders, such as polymyositis, are still of great interest and require better investigation to ensure appropriate management for each individual. Our experience suggests that aggressive immunosuppressive therapy might be a solution for severe post-COVID-related diseases. This literature review is provided in addition to the case report presented at the 29th European Paediatric Rheumatology Congress; the abstract is available online in the Proceedings of the 29th European Paediatric Rheumatology Congress.

## 1. Introduction

COVID-19-associated post-infectious clinical manifestations have been extensively studied since the pandemic. Various autoimmune manifestations after COVID-19 illness have been described, including reactive arthritis, hemolytic anemia, immune thrombocytopenia, cutaneous vasculitis, acute demyelinating disorders, multisystem inflammatory syndrome (MIS-C), and different connective tissue disorders, such as lupus and inflammatory myositis [1,2,3]. Studies have shown that these autoimmune conditions were significantly more common in patients with previous COVID-19 illness than in those who had not been infected. A study by Chang et al. included a large cohort of adult patients and identified that the incidence of any autoimmune disease was not only significantly higher after COVID-19 but also that the variety of new-onset illnesses was higher compared with other viruses [3]. For a significant period of time, infectious diseases have been recognized as potential triggers of autoimmune and autoinflammatory diseases, primarily through a mechanism known as molecular mimicry [4]. Severe acute respiratory syndrome coronavirus 2 (SARS-CoV-2) currently became one out of many infectious agents which have been identified as the potential triggers for these conditions [3,4].

The first wave of the pandemic showed that about 10% of COVID-19-infected patients developed muscle pain and different degrees of muscle weakness, as well as elevated creatin-kinase (CK) levels [5,6]. Post-COVID polymyositis is less discussed in the literature, but several cases have been described in adults [6,7,8,9,10]. However, data on pediatric cases are particularly scarce. Other authors have reported different cases of myositis post-COVID-19, either as part of MIS-C, autoantibody-positive inflammatory myopathies, or monomyositis, with no direct evidence linking them to SARS-CoV-2 [11,12,13,14]. In general, polymyositis is more frequently observed in young adults and is uncommon in children [15]. Muscle inflammation in polymyositis is characterized by proximal muscle weakness, which may progress over time, involving various muscle groups and presenting without skin manifestations [15]. It is important to consider a wide range of potential diagnoses, as polymyositis can imitate other myopathies and remains a diagnosis of exclusion [15].

In this article, we present a rare clinical case of severe post-COVID myositis in childhood and provide a literature review on post-COVID polymyositis.

## 2. Case Description

**Figure 1:**
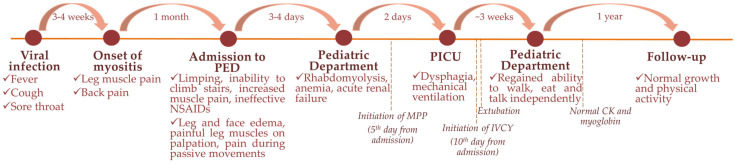
Development of the patient’s symptoms and complaints over different time points. Abbreviations: PED—pediatric emergency department; PICU—pediatric intensive care unit; NSAIDs—nonsteroidal anti-inflammatory drugs; MPP—methylprednisolone pulse; IVCY—intravenous cyclophosphamide; CK—creatin-kinase.

A 7-year-old boy was admitted to the pediatric department at the Hospital of Lithuanian University of Health Sciences Kaunas Clinics in Lithuania due to shin muscle pain and facial oedema. Physical exertion-related pain in the legs and episodic back pain started about one month before the admission to the pediatric department (Figure 1). Moreover, 3 weeks before the appearance of the aforementioned complaints, the boy had flu-like symptoms: febrile temperature for a few days, cough, runny nose, and sore throat. Subsequently, his condition deteriorated: the boy became irritable, lazy, refused to go to school due to intensive pain, limping, and inability to climb stairs. The effect of symptomatic treatment with nonsteroidal anti-inflammatory drugs (NSAIDs), such as ibuprofen and diclofenac, was insufficient. Regarding the worsening condition and pronounced thrombocytopenia (49 × 10^9^/L), the pediatrician referred the patient to the pediatric emergency department. Medical history indicated that he had overcome tonsillitis and various respiratory infections, without any chronic diseases or serious infections. He was vaccinated with the recommended vaccines, except against SARS-CoV-2. Clinical examination revealed swollen eyes, oedema of the legs, swollen hyperemic tonsils with whitish detritus, enlarged lymph nodes, painful calf and thigh muscles on palpation, and pain during passive movements, but the temperature was normal. The primary evaluation on the Childhood Myositis Assessment Scale (CMAS) [16,17] was 20 out of 52 points for muscle strength and function, indicating severe myositis. Blood tests revealed thrombocytopenia, elevated erythrocyte sedimentation rate (ESR), and anti-streptolysin O (ASO) titer but normal C reactive protein (CRP) and hemoglobin, as well as leukocyte count and formula (Table 1). Moreover, lower albumin, high liver enzymes (alanine transaminase (ALT), aspartate aminotransferase (AST), gamma-glutamyl transpeptidase (GGT)), and coagulation indicators (prothrombin time (PT) % activity, activated partial thromboplastin time (APTT)) were found (Table 1). In addition, abdominal ultrasound showed modest hepatosplenomegaly. Thus, the boy was hospitalized in the pediatric department.

**Figure 2:**
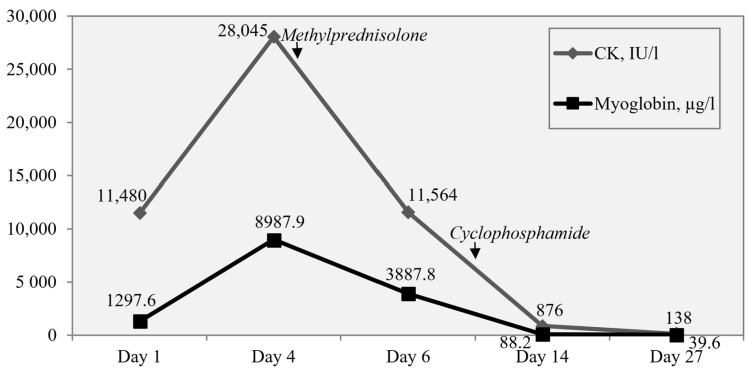
Timeline of muscle damage markers’ (myoglobin and creatin-kinase (CK)) levels and response to treatment.

Within four days from admission, muscle pain and weakness progressed to an inability to get out of bed. Increasing levels of CK 11,480→28,045 IU/L (normal range 54–269 IU/L) and myoglobin 1297.6→8987.9 µg/L (normal range 0–73 µg/L) were observed (Figure 2). Also, first signs of acute renal failure occurred: oliguria, oedemas, increase in serum creatinine by 44 µmol/L within 48 h (19→63 µmol/L (normal range 23–68 µmol/L)). Treatment was initiated with corticosteroids, choosing intravenous methylprednisolone pulses (MPP) of 20 mg/kg. However, within the next 24 h muscle weakness progressed to dysphagia (CMAS score lowering to 0 out of 52 points), the patient was intubated, and mechanical ventilation was started to avoid aspiration.

**Figure 3:**
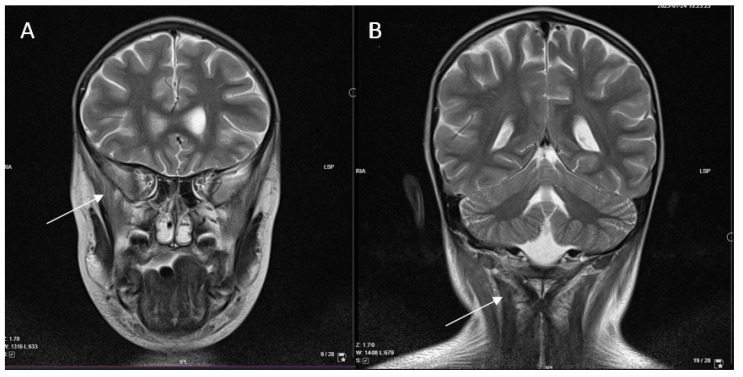
Head MRI, T2 sequence. Diffuse high signal in the *m. temporalis* (**A**) and neck muscles (**B**), as well as uneven accumulation of contrast medium in the *m. semispinalis* showing muscle oedema.

In order to determine the etiology of myositis, antibodies (IgG) against 11 antigens associated with myositis, IgG against 18 antigens associated with myopathies, antibodies against antigens of heart and skeletal striated muscles, muscle-specific tyrosine kinase, IgG against 23 nuclear antigens (ANA), anti-double-stranded deoxyribonucleic acid antibodies (anti-dsDNA), antineutrophil cytoplasmic antibodies (ANCA), and genetic acylcarnitine profile analysis were performed. All these tests were negative, as well as testing for Epstein–Barr virus (EBV), cytomegalovirus (CMV), a palette of 16 respiratory viruses, *Mycoplasma pneumoniae*, and *Chlamydia pneumoniae*. From all of the tested infection causes, only IgG against SARS-CoV-2 were found positive (140.3 BAU/mL, positive > 31.5 BAU/mL), confirming previous illness with the COVID-19 disease. As the boy remained afebrile, a polymerase chain reaction (PCR) test was not performed. Considering MIS-C as a possible diagnosis, a few characteristic but non-specific laboratory values, such as elevated ferritin (1000 µg/L; normal range 25–380 µg/L), thrombocytopenia, and elevated inflammatory markers (Table 1), were found. However, there were not enough criteria for MIS-C, as clinical manifestation was limited to muscle weakness without fever, rash, gastrointestinal symptoms, or cardiac damage. Moreover, thyroid function was normal (thyroxine 10.2 pmol/L; normal range 10–22 pmol/L; thyrotropin 1.78 mU/L; normal range 0.28–4.3 mU/L), disproving polymyositis-like syndrome. In parallel, immunodeficiency, hemophagocytic lymphohistiocytosis (HLH) or neurological disorders were rejected by a multidisciplinary team. In addition, head MRI (magnetic resonance imaging) and CT (computed tomography) scan of the chest, abdomen, and pelvis disproved a possible paraneoplastic process but showed diffuse oedema of the neck and upper chest muscles (Figure 3).

In a peak of disease severity, several complications, such as anemia (Hb 65 g/L), hypokalemia 2.75 mmol/L (normal range 3.4–5.4 mmol/L), and acute tracheitis, confirmed by massive growth of *Streptococcus pneumoniae* and *Staphylococcus aureus* in tracheal secretion, appeared. All of these were resolved by adding antibiotics, applying infusions, and erythrocyte mass transfusion.

After the third day of corticosteroid pulse therapy, the patient’s condition started to improve—CK decreased by 2.7 times and myoglobin by 2.5 times from the peak (Figure 2)—and the patient was extubated in 4 days, continuing feeding through a nasogastric tube. MPPs were administered for a total of 5 days and then switched to oral prednisone 2 mg/kg, tapering during the next 6 months. Rapid progression to a life-threatening condition for this patient and insufficient effect after MPP led to thinking about an autoimmune-driven process, demanding continuous immunosuppression. Due to persistent dysphagia and dysphonia (CMAS score after 5 days of MPPs was 8 out of 52 points) and major organ involvement (kidneys), intravenous cyclophosphamide was added at a dose of 750 mg/m^2^ every 4 weeks. Clinical condition and laboratory tests improved 3 weeks after the initiation of corticosteroids and cyclophosphamide—myoglobin and CK normalized, inflammatory markers were normal, and renal function was restored. Physiotherapy and logotherapy together with continuing immunosuppressive therapy and analgesics conditioned the ability to walk, eat, and talk independently, and the boy was discharged after one month of inpatient treatment. Gradual improvement was observed over the next few months on treatment with oral prednisone and cyclophosphamide. Cyclophosphamide pulses were stopped after the fourth (total cumulative dose 3000 mg/m^2^), leaving low-dose prednisone for the next 2 months. Without treatment, the patient demonstrated normal growth and physical activity during the follow-up of one year (Figure 1).

## 3. Discussion

In this manuscript, we report a case of severe pediatric polymyositis after previous COVID-19 illness. While all of the available-to-measure autoantibodies for myopathies were negative, IgG against SARS-CoV-2 were found positive, confirming its likely association with polymyositis. A few similar cases in adulthood, published by different authors [6,7,8,9,10], are compared in Table S1 (Supplementary Materials). Although the data provided in individual cases differ, most cases were antibody negative, the time between infection (determined by clinical picture or PCR test) and myositis varied from 1 to 6 months, and all patients responded to immunosuppressive treatment mainly with glucocorticoids. No deaths or long-term sequelae were reported. Descriptions of the cases range from myositis to myopathies, including necrotizing autoimmune myositis (NAM).

**Inflammatory myopathies and COVID-19.** Overall, five types of inflammatory myopathies are known: dermatomyositis (DM), polymyositis, NAM, anti-synthetase syndrome-overlap myositis (anti-SS-OM), and inclusion-body myositis (IBM) [5]. The classification of polymyositis within idiopathic inflammatory myopathies has become a subject of discussion due to the possibility of regrouping it as other forms of inflammatory myopathies [18]. Juvenile DM is the predominant form of idiopathic inflammatory myopathy observed in pediatric patients, accounting for approximately 75% of cases [18]. Mohavedi and Ziaee noted that the number of juvenile DM cases in Iran had grown during the pandemic and hypothesized that these newly diagnosed cases were not only triggered by previous SARS-CoV-2 infection but also as a late consequence of COVID-19 disease or dermatomyositis-like autoimmune syndrome, resembling the immunopathogenesis of better-known Kawasaki-like MIS-C [13]. In contrast to DM, NAM is less common, occurring in up to 21% of juvenile cases, while IBM and anti-SS-OM are rarely observed in children [18]. A study by Romero-Sanchez et al. examining neurological complications in 841 hospitalized patients with COVID-19 revealed that myopathy was observed in 3.1% of these individuals [19]. Notably, the majority of myopathy cases were associated with critical illness and prolonged hospitalization in the intensive care unit, which might be critical in association with rhabdomyolysis [19]. In a review by Saud et al., of the 22 case reports found on various types of COVID-19-related myositis, the youngest patient was 26 years old and the highest CK level detected was 42,670 IU/L [20]. However, post-COVID myositis distinct from the acute infection and without acute COVID-19 manifestations seems to be quite rare, especially in children. To the best of our knowledge, no such cases have been reported in the literature, except several reports of rhabdomyolysis as the main presentation of acute COVID-19 disease in adolescents [21,22,23,24].

**Virus-induced myositis.** Nevertheless, virus-induced myositis is well known and has been widely reported. Influenza is the most prevalent infection leading to rhabdomyolysis, accounting for approximately one-third of cases associated with viral infections [25]. Retroviruses, particularly human immunodeficiency virus (HIV), have been found to develop polymyositis or inclusion-body myositis, while retroviral antigens have been detected within certain endomysial macrophages and lymphoid cells, suggesting indirect immune-mediated muscle damage [5,26]. Based on molecular investigations, to date, none of the viruses associated with potential myositis triggers have demonstrated direct infection of muscle fibers [5]. However, viruses stimulate muscle fiber invasion with pro-inflammatory cytokines, mediated by macrophages or clonally expanded T cells specific to the virus [5].

**COVID-19-induced myositis.** SARS-CoV-2-induced myopathy is arising as a novel condition. SARS-CoV-2 has been observed to cause hyperCK-emia, suggestive of NAM according to its pattern [5]. However, recent studies have shown that elevated CK levels are not always present, as there is wide diversity in the forms of COVID-19-associated myositis reported in adults (classical DM with rashes, rhabdomyolysis, or isolated paraspinal myositis) [20]. Moreover, it is sometimes unclear whether SARS-CoV-2 infection was the cause or only a trigger to induce myositis, especially when muscle-specific autoantibodies are found [27].

**Pathogenesis of COVID-19-induced myositis.** Scientists offered several theories of pathogenesis of COVID-19-associated myositis. These hypotheses include the possibility of direct muscle damage caused by the virus entering through the angiotensin-converting enzyme (ACE2) receptor, which is present in skeletal muscles [20]. However, the lack of data on the ability of viruses to enter myocytes directly, as well as autopsy studies, which did not detect SARS-CoV-2 protein within muscle cells, makes this mechanism less likely [18]. Another hypothesis suggests that polymyositis may be triggered by an autoimmune response, resulting in innate and adaptive immune hyperactivation [20]. This concept may also be applicable to post-viral myositis.

**Definition of post-COVID myositis.** Viral infections can induce a broad range of muscle disorders, from myalgia to myositis and rhabdomyolysis (defined as CK elevation by five times above the upper limit) [25]. There are several terms used by different authors to describe virus-induced conditions. Firstly, acute viral myositis is typically described as occurring during recovery from acute infectious disease (on average on the third day of fever or other symptoms) and lasts for about a week [26]. Mostly it has a benign and self-limiting course [26]. Secondly, the term post-viral myositis is usually used for the conditions that last for approximately one month [7], albeit the gap between acute infection and the onset of myositis is not strictly defined. In most cases, the reported symptom-free period ranges from several weeks to months after acute infection. However, as there are no clear definitions agreed upon universally, other authors describe post-viral myositis as muscle inflammation, starting 3–7 days after the flu-like condition (like in cases of acute viral myositis) with variable duration of myositis symptoms, depending on case complexity [13]. Different reported post-COVID conditions begin several days to 6 months after viral illness [2]. Some of the reported cases could fit the description of acute virus-induced conditions, while others fit the description of post-viral pathologies. Additionally, some but not all of them can be defined as having a long COVID syndrome. According to the definition of post-COVID condition (long COVID), currently provided by the World Health Organization, adverse symptoms of acute SARS-CoV-2 infection persist or arise within 3 months but last for at least 2 months, if no alternative diagnosis is found [28]. Although various definitions related to post-COVID sequelae have been proposed by different initiative groups (a summary can be found in the review by Munblit et al. [29]), post-COVID myositis is still not clearly defined. The clinical manifestation of muscle injury in our presented case started after a previous flu-like infection with a 3-week symptom-free period. Positive SARS-CoV-2 IgG antibodies were detected, suggesting that this virus could be the causative agent. According to the literature, elevated IgG levels remain for 12 months in 75% of COVID-19 survivors [30], and one study revealed that approximately half experienced a decrease in IgG levels 10 months after recovery [31]. Quantitative IgG analysis in a C19.CHILD Hamburg cohort demonstrated faster decreasing antibody levels in children compared to adults: the mean serum IgG level in children was ~160 AU/mL initially, ~100 AU/mL after 3 months, and ~60 AU/mL after 6–9 months [32]. As our patient had significant IgG elevation, recent SARS-CoV-2 infection was considered as a possible predisposing factor. Altogether, our patient fits both post-viral myositis and post-COVID condition descriptions, suggesting a post-COVID myositis definition of myositis arising several weeks to months after the resolution of symptomatic or asymptomatic acute SARS-CoV-2 infection, if no alternative diagnosis exists.

**Diagnostics.** In general, the clinical manifestation of inflammatory myopathies may vary from proximal muscle weakness to severe involvement of the neck extensor and pharyngeal muscles resulting in head drop and dysphagia [5], as observed in our case. The respiratory musculature may be impaired in complex cases [5]. Our case could fit a seronegative immune-mediated necrotizing myopathy (or NAM). However, only histological examination could confirm this. Pedraz et al. denoted that very few cases of childhood seronegative NAM have been published, and those ones were associated with longer duration and an inclination to be more resistant to initial therapy than other inflammatory myopathies [33].

The diagnosis of different myopathies is based on clinical signs and symptoms in combination with an increase in serum muscle enzymes, histological confirmation, and specific autoantibodies, distinguishing different types of inflammatory myopathies [5,18]. Although histological data specifically on post-COVID polymyositis are scarce, other authors have reported characteristic findings for muscle inflammation: endomysial lymphocytic infiltrates, skeletal muscle atrophy, and its replacement by fibroadipose tissue [9]. Additional examinations include electroneuromyography (ENMG) and muscle MRI, which can help exclude other causes of myopathy and evaluate disease activity and distribution of affected muscles [5,18]. In the case we described, painful procedures, such as ENMG and muscle biopsy, were not performed because of the patient’s young age. In addition, biopsies in children are currently being replaced by MRI of various areas of the body. Data on the relevance of imaging techniques in official guidelines, such as the American College of Rheumatology and European League Against Rheumatism (ACR/EULAR), are lacking [34]. However, they no longer insist on muscle biopsy for the diagnosis of inflammatory myopathy, when there is a clear clinical presentation with pathological imaging findings, specific autoantibodies, and laboratory markers [34]. Studies have demonstrated MRI sensitivity for idiopathic inflammatory myopathies of 90% and specificity of 66–86% [35]. Malarte et al. acknowledged that MRI is an excellent tool for diagnostic and follow-up purposes but cannot distinguish subtypes of inflammatory myopathies as muscle biopsy can [35]. Although a biopsy was not performed in our case, we relied on MRI and CT scans, which revealed muscle oedema typical for myositis.

**Polymyositis-like syndrome.** Regardless of the already discussed post-viral myositis and true polymyositis, a polymyositis-like syndrome should be considered in terms of differential diagnosis. Likewise, it presents muscle weakness and CK elevation [36]. The most common cause—hypothyroidism—can be detected by thyroid function tests and successfully treated with thyroxine [36]. In our case, the patient’s thyroid function was normal.

**Limitations of our case study.** Although our case report has some limitations for clear myositis entity description (ENMG and muscle biopsy were not performed) and confirmation of SARS-CoV-2 by PCR test during acute respiratory infection prior to myositis, the diagnosis of post-COVID polymyositis was made taking into account clinical manifestation, positive SARS-CoV-2 antibodies, and by disproving other possible etiologies of myositis (autoimmune, infectious, paraneoplastic syndrome, etc.). The patient remained in favorable condition during the untreated follow-up period, suggesting an acute rather than chronic process. The symptoms started with shin muscle pain, which is a typical localization of muscle damage in post-viral myositis. Eventually, other muscle groups were affected by acute rhabdomyolysis, which is also a common sign of post-viral myositis.

## 4. Conclusions

In conclusion, this clinical case reveals a relatively new and poorly studied post-infectious variant of severe polymyositis in a child with previous COVID-19 infection. This is an example of successful immunosuppressive therapy, which led to a great improvement in the patient’s clinical condition and laboratory markers. As the data about this condition in children are lacking, more clinical experience and further studies are necessary to explain the mechanisms of post-COVID-induced autoimmune pathologies and find the most appropriate management solution.

## Figures and Tables

**Table 1:** Initial blood test results (emergency department).

	Result	Normal Range
Thrombocytes	60 × 10^9^/L	184–534 × 10^9^/L
ESR	34 mm/h	0–11 mm/h
CRP	5 mg/L	0–5 mg/L
ASO titer	251.2 U/mL	0–150 U/mL
Albumin	30.1 g/L	35–50 g/L
ALT	2059 IU/L	7–55 IU/L
AST	674 IU/L	8–60 IU/L
GGT	220 IU/L	3–22 IU/L
PT % activity	134%	70–130%
APTT	40.7 s	28–38 s

Abbreviations: ESR—erythrocyte sedimentation rate; CRP—C reactive protein; ASO—anti-streptolysin O; ALT—alanine transaminase; AST—aspartate aminotransferase; GGT—gamma-glutamyltranspeptidase; PT prothrombin time; APTT—activated partial thromboplastin time.

## Data Availability

The original contributions presented in this study are included in the article/Supplementary Materials; further inquiries can be directed to the corresponding author.

## Supplementary Materials

The following supporting information can be downloaded at: https://www.mdpi.com/article/10.3390/children11081011/s1, Table S1: Comparison of publications on post-COVID myositis.

## Abbreviations

ACE-2—angiotensin-converting enzyme 2; ALT—alanine transaminase; ANA—antibodies against nuclear antigens; ANCA—antineutrophil cytoplasmic antibodies; Anti-dsDNA—anti-double-stranded deoxyribonucleic acid antibodies; Anti-SS-OM—anti-synthetase syndrome-overlap myositis; APTT—activated partial thromboplastin time; ASO—anti-streptolysin O; AST—aspartate aminotransferase; CK—creatin-kinase; CMAS—Childhood Myositis Assessment Scale; CMV—cytomegalovirus; COVID-19—coronavirus disease 2019; CRP—C reactive protein; CT—computed tomography; DM—dermatomyositis; EBV—Epstein–Barr virus; ENMG—electroneuromyography; ESR—erythrocyte sedimentation rate; GGT—gamma-glutamyl transferase; Hb—hemoglobin; HIV—human immunodeficiency virus; HLH—hemophagocytic lymphohistiocytosis; IBM—inclusion-body-myositis; IFN—interferon; IgG—immunoglobulin G; MIS-C—multisystem inflammatory syndrome in children; MPP—methylprednisolone pulse; MRI—magnetic resonance imaging; NAM—necrotizing autoimmune myositis; NSAIDs—nonsteroid anti-inflammatory drugs; PCR—polymerase chain reaction; PT—prothrombin time; SARS-CoV-2—severe acute respiratory syndrome coronavirus 2.
