# Supplementary material for: Severe Post-Viral Polymyositis after COVID-19 in Childhood: A Case Report and Literature Review
Source: Children (Basel). 2024 Aug 20;11(8):1011. doi: 10.3390/children11081011 (PMC11352915; doi:10.3390/children11081011)
Supplement: Supplementary file 1 [file children-11-01011-s001.zip › children-3125302-supplementary.pdf]

Supplement 1

Table S1. Comparison of publications on post-COVID myositis

First author	Patient	CK level (IU/l)	Autoantibodies	Acute infection & SARS-CoV-2 PCR	SARS-CoV-2 antibodies	Time between infection and myositis	Diagnostics	Treatment
Veyseh et al., 2021 [6]	57 years, female	15000	*Positive:*<br>ANA 1:320, anti-Smith (low titer)<br>*Negative:* anti-PL, anti-Jo-1, anti-SRP, anti-HMGCR, other myositis-related	Mild respiratory tract infection<br><br>Positive test	Positive, level not mentioned	1 month;<br>4 months (repeated)	Biopsy-confirmed NAM	Prednisone 1mg/kg/d, tapering over 3 months
Anthony et al., 2022 [7]	58 years, male	274<br>(n 44-196)	*Negative:*<br>ANA, RF, anti-CCP	Mild respiratory tract infection<br><br>PCR test not mentioned	Not mentioned	6 months	Biopsy and ENMG not performed;<br>Brain MRI to exclude multiple sclerosis	High dose prednisone
Shetty et al., 2022 [8]	47 years, male	644	Not mentioned	Severe respiratory tract infection<br><br>Positive test	Not mentioned	3 months	MRI-confirmed myositis	Indomethacin 75mg/d and prednisone 50mg/d (with tapering after 2 weeks) for 3 weeks
Amin et al., 2022 [9]	52 years, female	2225	*Negative:*<br>ANA, RF, anti-CCP	Mild respiratory tract infection<br><br>Positive test	Not mentioned	4 months	ENMG;<br>MRI;<br>Biopsy-confirmed inflammatory myopathy	Prednisone 60mg/d with tapering over 4 months and AZA 50mg x2/d for 4 weeks
Lokineni et al., 2021 [10]	51 years, male	27000	*Negative:* anti-Mi-2, anti-Ku, anti-PM-Scl-100, anti-PM-Scl-75, anti-Jo-1, anti-SRP, anti-PL-7, anti-PL-12, anti-EJ, anti-OJ, anti-Ro-52, ANA, anti-dsDNA, anti-Smith, anti-RNP, anti-SSA, anti-SSB, ANCA, anti-MPO, anti-PR-3	Severe COVID-19 pneumonia<br><br>Positive test	Not mentioned	3 months	Biopsy-confirmed NAM	Prednisone 60mg/d for 3 weeks, then tapering and AZA 150mg/d (2mg/kg)

Abbreviations: CK – creatin-kinase; ANA – antinuclear antibodies; anti-PL – antiphospholipid; anti-Jo-1 – anti-histidyl transfer RNA synthetase; anti-SRP – anti-signal recognition particle; anti-HMGCR – anti-3-hydroxy-3-methylglutaryl-CoA reductase; anti-CCP – anti-cyclic citrullinated peptide; RF – rheumatoid factor; anti-Mi-2 – anti-Mi-2 protein; anti-Ku – anti-DNA-binding protein Ku,; anti-PM-Scl-100 – anti-polymyositis/ systemic sclerosis-100; anti-PM-Scl-75 – anti-polymyositis/ systemic sclerosis-75; anti-PL-7 – anti-threonyl-tRNA synthetase; anti-PL-12 – anti-alanyl-tRNA synthetase; anti-EJ – anti-aminoacyl-tRNA synthetase; anti-OJ – anti-isoleucyl-transfer RNA synthetase; anti-Ro-52 – anti-tripartite motif-containing protein 21; anti-dsDNA – anti-double stranded deoxiribonucleoacid; anti-RNP – anti-ribonucleoprotein; anti-SSA – anti-Sjögren's syndrome type A; anti-SSB – anti-Sjögren's syndrome type B; anti-MPO– anti-myeloperoxidase; anti-PR-3 – anti-proteinase-3; PCR - polymerase chain reaction; NAM - necrotizing autoimmune myositis; ENMG – electroneuromyography; MRI – magnetic resonance imaging; AZA – azathioprine; MMF – mycophenolate mofetil
